# The use of Subject Matter Experts in Validating an Oral Health-Related Quality of Life measure in Korean

**DOI:** 10.1186/s12955-015-0335-0

**Published:** 2015-09-04

**Authors:** Jaesung Seo, Michael MacEntee, Mario Brondani

**Affiliations:** Schulich School of Medicine and Dentistry Western University, Ontario, Canada; Department of Oral Health, Sciences. Division of Prosthodontics and Dental Geriatrics, University of British Columbia, Vancouver, Canada; Department of Oral Health Sciences. Division of Preventive & Community Dentistry, and Prosthodontics and Dental Geriatrics, University of British Columbia, JBM 122/2199 Wesbrook Mall, Vancouver, BC V6T 1Z3 Canada

## Abstract

**Objectives:**

This paper aimed to employ subject matter experts (SMEs) to assess the extent to which the Korean version of the short-form of the OHIP (OHIP-14 K) is culturally valid and equivalent in Korean.

**Methods:**

We approached 17 bilingual Korean SMEs from which 10 independently rated the clarity, relevance, and cultural equivalence of the OHIP-14 K. SME's varied between 10 and 41 years of clinical experience and were mostly males (# 7). We used Item-level Content Validity Index (I-CVI) to gauge the proportion of SMEs who considered the content of OHIP items (e.g., instruction, response format, etc.) to be culturally valid. We also performed additional analysis to determine the level of agreement between the SMEs.

**Results:**

The experts rated most of the items to be clear (S-CVI = 0.93) while having difficulties in assigning relevance of the questions to the expected domains (S-CVI = 0.42). Moreover, considerable disagreement existed among the experts in regard to the relevance (Kfree = 0.19 to 1.00) and the cultural equivalence indexes (ADM = 0.36 to 0.96). The content of the OHIP-14 K for the most part clearly reproduced the language of the original OHIP-14. However, experts disagreed on the relevance and conceptual equivalence of the OHIP-14 K for a Korean population.

**Conclusions:**

Patient-oriented outcome measures such as the OHIP can be used across cultures once there are indeed assessing the same domains and constructs of interest. The CVI technique seems to be an alternative tool for evaluating content validity and equivalency of an OHQoL measure. A more refined, culturally relevant version of OHIP-14 K was proposed although there is no available data yet to support a better score validity, reliability and responsiveness of this proposed version.

## Introduction

Oral health-related quality of life (OHQoL) represents a psychological *construct* defined as self-reports pertaining to the functional, psychological and social impacts of oral problems on quality of life [1]. Many OHQoL measures are available worldwide for exploring the self-perceived status of oral health via surveys and for comparing before and after treatment outcomes via clinical trials, for example [2–6]. The Oral Health Impact Profile (OHIP) has been the most used self-reported measure of OHQoL based on the International Classification of Impairment, Disability and Handicap as interpreted to oral health by David Locker in 1988 [7–9]. It consists of 49 questions representing seven domains (Table 1) assessed by a 5-point Likert response scale (“very often”; “fairly often”; “occasionally”; “hardly ever”; or “never”, with an optional “don’t know”). The OHIP-14 is a shortened version of the OHIP-49 reduced through regression and item-impact analysis to 14 questions [10].

**Table 1:** The theoretical domains and functional items of the original short form of the Oral Health Impact Profile (OHIP-14)

Theoretical Domains	OHIP-14 Item
Functional Limitation	Q1. Have you had trouble pronouncing any words because of problems with your teeth, mouth or dentures?
	Q2. Have you felt that your sense of taste has worsened because of problems with your teeth, mouth or dentures?
Pain & Discomfort	Q3. Have you had painful aching in your mouth?
	Q4. Have you found it uncomfortable to eat any foods because of problems with your teeth, mouth or dentures?
Psychological Discomfort	Q5. Have you been self-conscious because of your teeth, mouth or dentures?
	Q6. Have you felt tense because of problems with your teeth, mouth or dentures?
Physical Disability	7. Has your diet been unsatisfactory because of problems with your teeth, mouth or dentures?
	Q8. Have you had to interrupt meals because of problems with your teeth, mouth or dentures?
Psychological Disability	Q9. Have you found it difficult to relax because of problems with your teeth, mouth or dentures?
	Q10. Have you been a bit embarrassed because of problems with your teeth, mouth or dentures?
Social Disability	Q11. Have you been a bit irritable with other people because of problems with your teeth, mouth or dentures?
	Q12. Have you had difficulty doing your usual jobs because of problems with your teeth, mouth or dentures?
Handicap	Q13. Have you felt that life in general was less satisfying because of problems with your teeth, mouth or dentures?
	Q14. Have you been totally unable to function because of problems with your teeth, mouth or dentures?

Adapting a QoL measure like the OHIP from English to another language offers the possibility for cross-cultural comparisons [11–13] once assumed that its conceptual foundation in the ICIDH and Locker’s model is accepted at face value, and that the concepts and domains addressed are readily transferable between cultures. However, cultural environment strongly influence personal identity and how people consider, interpret and cope with chronic diseases and disorders [5, 6]. The OHIP has been translated into more than 30 languages via various methodologies, but not without challenges to achieve validity and equivalency to the original Australian-English version [6]. Despite reservations about the validity of the OHIP’s conceptual foundations [5,6] and domains’ structure [14], Bae et al. (2007) adapted the OHIP-14 to Korean (OHIP-14 K) using bilingual translators without fully validating its concepts; yet with assumptions that it is a valid and equivalent translation [15]. The objective for this study was to assess the content validity and cultural equivalence of the Korean version of the OHIP-14 K with the assistance of bilingual subject matter experts (SMEs).

## Methods

### Selection of Subject Matter Experts - Ethics, consent and permissions

Ethical approval was obtained from the University of British Columbia Behavioral Research Ethics Board # H10-01023. The selection of expert participants was based on the 2009 College of Dental Surgeons of British Columbia directory by purposefully searching for practicing dentists with Korean first and family names. The participants included those who were practicing in Vancouver at least three times a week for a minimum of 10 years, understood and used both the Korean and English, and were willing to participate in this study. Although the number of recruited SMEs varies [16, 17], Beck and Gable (1986) suggested a minimum of 10 participants to yield acceptably consistent responses and to avoid chance agreement (Table 2). [18] We have identified 20 eligible participants; 17 were successfully contacted as the other 3 could not be reached at their listed addresses. From the 17 who were contacted, 10 volunteered to participate while the others refused to take part in the study because of their busy schedules or lack of interest..

**Table 2:** Background information of the subject matter experts

SME	Gender	Place(s) of Graduation	Clinical Experience (yrs)	Education Background	Location
1	M	University of British Columbia; University of Temple; University of California, San Francisco	15	DDS, PhD, AEGD^1^	Burnaby, BC
2	M	Seoul National University; University of British Columbia	26	DDS/MD	Burnaby, BC
3	M	Undisclosed	30	DMD	Coquitlam, BC
4	M	Yonsei University; University of Manitoba; University of British Columbia	19	DMD, MSc, PhD	Burnaby, BC
5	F	Southwestern University	14	DMD	North Vancouver, BC
6	M	University of London; Seoul National University; Korea University	41	BDS, DMD, MSc, PhD	Vancouver, BC/ Seoul, Korea
7	F	University of British Columbia	10	BDS, DDS	Burnaby, BC
8	M	University of Manitoba	15	DDS	North Vancouver, BC/ Lancaster, CA
9	F	University of Pennsylvania; Columbia University	12	DDS, MA	Coquitlam, BC
10	M	Korea University; University of British Columbia	26	DDS (SNU), DMD (UBC), MSD, PhD, PPD	Burnaby, BC

^1^Advanced Education in General Dentistry

### Consent to publish

Consent to publish was obtained from the participants as they signed off on the following statement on the written consent form: “*Your signature indicates that you consent to participate in this study. You are willing to have your interview audio-taped and give permission for the principal investigator to use the information you are providing anonymously as part of a publication focused on the same issue.*”

Content validation (CV) began with the construction and administration of a questionnaire to gather quantitative and qualitative information on the clarity, cultural equivalency and relevance of the OHIP [14,18], and was pilot-tested for clarity by two local practicing Korean dentists outside the group of 10 who participated in the study. They both recommended that the WHO’s definitions of impairment, disability, and handicap (1980) be appended as background material to consult.

We then designed a questionnaire with three comprehensive subscales for SMEs to judge: 1) the content validity of OHIP-14 K in terms of the technical quality of the items, instructions, and response formats; 2) the relevance of the content to the ICIDH theoretical domains of Locker’s model; and 3) the cultural equivalency of the translation to the intent of the original OHIP-14. On the recommendation of Lynn [16], the CV questionnaire acquired a 4-point ordinal Likert scale as a response format without neutral ground.

In the clarity index, the SMEs were asked to rate the clarity of the instructions, items, and response format using the following Likert scale: 0 = not at all clear, 1 = somewhat clear, 2 = mostly clear, and 3 = very clear [19]. They were instructed to identify comprehension problems with the OHIP-14 K due to, for example: vague wording, ambiguous language, double-barrelled questions, and so on.

In the cultural equivalence index, the SMEs were asked to evaluate the semantic, colloquial, experiential and conceptual equivalence, rather than linguistic or literal equivalence of the translation using the scale: 0 = not at all equivalent, 1 = somewhat equivalent, 2 = mostly equivalent, and 3 = equivalent. They were encouraged to also comment generally on the translation with suggestions for deletions, additions and modifications.

In the relevance index, the degree to which the OHIP-14 K items appropriately sampled the theoretical domains of OHIP (e.g., functional limitation, social disability, etc.) was assessed. The SMEs were asked to identify for each item the most appropriate theoretical domain of Locker’s model.

All information, including the various types of cultural equivalence, was written and verbally conveyed to the SMEs when meeting with the first author (JS). After this introductory briefing, the SMEs signed the informed consent form and received a CV questionnaire with the instructions and the OHIP-14 in both Korean and English. They answered the CVI questionnaires individually, independently, and at their own convenience. The SMEs were again visited within 30 days where the questionnaires were collected.

## Data analysis

The Likert responses were analyzed using SPSS® (IBM Corp, Version 19.0. Armonk, NY: IBM Corp). The clarity of the OHIP-14 K was rated for relevance and equivalence both for the entire scale (S-CVI) and individually for the instructions, response format, event frequency, and items (I-CVI). The I-CVIs were calculated as the proportion of SMEs who endorsed the validity of each scale (i.e., ratings of 3 or 4 on the 4-point Likert scale) as suggested by Beck and Gable [18].

$$ \mathrm{C}\mathrm{V}\mathrm{I} = \frac{\mathrm{Number}\ \mathrm{of}\ \mathrm{responses}\ \mathrm{a}\mathrm{s}\ ``3\ \mathrm{or}\ ``4"\ }{\mathrm{Total}\ \mathrm{number}\ \mathrm{of}\ \mathrm{responses}} $$

**Fig. 1:**
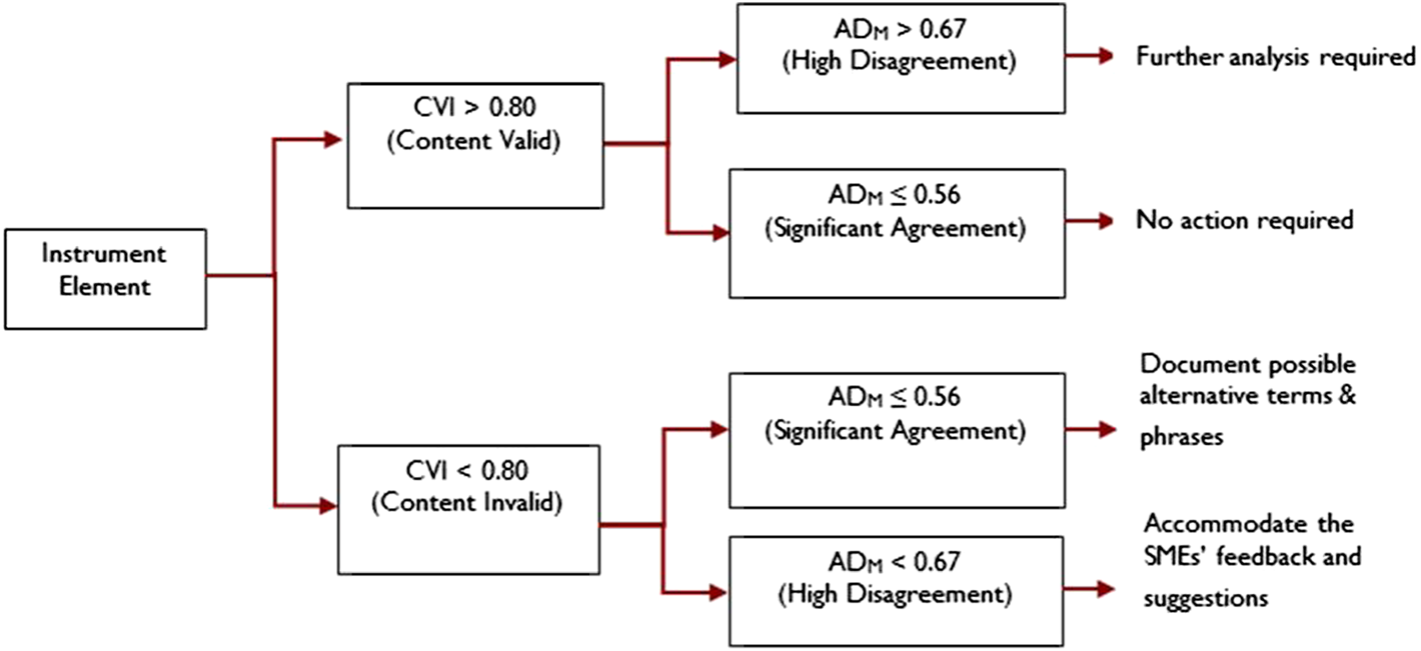
Possible outcomes of content validation study of OHIP-14k

The AD_M_ measures multi-rater disagreement in the scale’s units and uncovers hidden disagreement in dichotomous data. AD_M_ for the clarity and cultural equivalence indices was calculated as the sum of the differences between individual ratings and the mean in absolute values divided by the total number of ratings. A lower AD_M_ value indicated stronger agreement between SME-ratings because the AD_M_ is dispersed around the mean, while disagreement might lead to revisions on the instrument (Fig. 1).

We used non-parametric Kappa statistics for the analysis of data generated by the relevance index in which the most representative theoretical domain was selected as suggested by Slocumb and Cole [20]. Kappa values below the cut-off 0.4 were considered to be poor agreement [20] and prompted further examination of the SMEs’ comments. The Scale-level CVI was  expressed as the percentage of items whose I-CVI values were equal to or greater than the minimally acceptable CVI of 0.78; it provided information on the proportion of elements requiring revisions until the S-CVIs was equal to or greater than 0.80 to confirm the validity of the scale [21].

## Results

### Clarity of OHIP-14 K Elements

With the exception of the response format, all 16 elements (including items and OHIP instructions) had I-CVI values greater than 0.80, indicating adequate levels of clarity (Table 3). The deviation from the mean index (AD_M_) across all elements was below the critical value of 0.56, suggesting that homogeneity in SME ratings was unlikely to have been achieved by chance. For the most part, the OHIP-14 K was judged to be clear (S-CVI = 0.93, AD_M_ ≤ 0.56). The only element whose CVI value felt below the acceptable level was the response format (I-CVI = 0.7), which was considered to be vague. SME’s recommended changing the Korean words “*maewoo*” (very) to “*maewoo jaju*” (very often), and “*guhee*” (hardly) to “*guhee junhyu*” (hardly ever). In addition, three other SMEs commented that the Korean translation of the OHIP-14 asks about symptoms that overlap significantly with systemic illnesses such as depression, so it could potentially have diverse interpretations. Consequently, they recommended that the specific oral health context be expressed in every question by including the phrase “*because of problems of your mouth, teeth or dentures*” as in the original English version.

**Table 3:** Item-level content Validity Index (I-CVI) and Average Deviation from the Mean Index (AD_M_) of OHIP-14 K instruction, response format, event frequency, and items in the Clarity and Equivalence Indices

	Clarity Index^1^		Equivalence Index^2^	
Original Scale Element *(back-translation of OHIP14-K)*	I-CVI	AD_M_	I-CVI	AD_M_
Instruction	0.9	0.38	0.3	0.55
Response Format	0.7	0.34	0.8	0.63
Event Frequency^a^	1.0	0.40	0.6	0.54
1. Trouble pronouncing any words	1.0	0.40	0.6	0.96
(*Discomfort from not being able to pronounce well*)				
2. Sense of taste has worsened	1.0	0.38	0.7	0.64
(*Sense of taste was worse than before*)				
3. Painful aching in your mouth	1.0	0.40	0.4	0.70
(*Pain in the tongue, sublingual, cheeks, palate, etc.*)				
4. Uncomfortable to eat any foods	1.0	0.39	0.7	0.60
(*Uncomfortable to have meal due to painful or uneasy problems of the mouth*)				
5. Self-conscious	0.9	0.38	0.4	0.56
(*Reluctant to meet others because of shame*)				
6. Felt tense	0.9	0.38	0.6	0.54
(*Paid attention to*)				
7. Unsatisfactory diet	1.0	0.40	0.9	0.48
(*Dissatisfied meals*)				
8. Meals interrupted	1.0	0.40	0.8	0.36
(*Interrupted during meals*)				
9. Difficult to relax	1.0	0.39	0.8	0.36
(*Difficulties resting comfortably*)				
10. A bit embarrassed	0.9	0.39	0.9	0.36
(*Embarrassed or perplexed*)				
11. A bit irritable with other people (*Get angry easily at others*)	0.9	0.37	0.5	0.70
12. Difficulty doing your usual jobs	0.9	0.37	0.9	0.56
(*Difficult to do normal jobs*)				
13. Life in general was less satisfying	0.9	0.37	0.8	0.54
(*Life less satisfying than before*)				
14. Totally unable to function	0.8	0.36	0.6	0.72
(*Psychologically, physically and socially cannot at all do one’s share*)				

^a^Two missing values from two questionnaires (SME 7 and 8) were imputed with the item mean value for that scale element.

^1^Clarity Index S-CVI = 0.93 ^2^ Equivalence Index S-CVI = 0.50

### Cultural Equivalence between OHIP-14 and OHIP-14 K

The SMEs were instructed to evaluate the cross-cultural equivalence between the OHIP-14 in English (OHIP-14E) and OHIP-14 K. Seven elements – which included the response format and questions 7, 8, 9, 10, 12, and 13 – were deemed content valid (CVI > .80), all with acceptable agreement (AD_M_ ≤ 0.56). On the contrary, items including the instructions, the frequency scale, and questions 5 and 6 felt below the minimally acceptable CVI value with statistically significant agreement (I-CVI < 0.80, AD_M_ ≤ 0.56). While neither significant agreement nor disagreement was observed for items 2 and 4, a high level of disagreement was noted for items 1, 3, 11, and 14 with regard to cultural equivalency. For example, the SMEs were divided over the cultural equivalency of the Korean translation of question 1: *trouble pronouncing words* (AD_M_ = 0.96). Four SMEs suggested that *having trouble pronouncing words* had a different meaning than the Korean translation *discomfort from not being able to pronounce well* as offered in the current OHIP-14 K. The suggested revisions for question 1 included “*baleumeul mothaesuh himdeushinjuk*” (having difficulties to pronounce [any words]). The experts also disagreed over the equivalency of question 14 (AD_M_ = 0.72), “*totally unable to function*,” which was translated into “*jungshinjeok, shinchejeok, sahoejeokeuro junhyuh jemokeul halsu upsutdun jeok*” (totally unable to do one’s share psychologically, physically, and socially).

### Relevance of OHIP-14 K Domains

Table 4 shows the distribution of SME’s responses when asked “*Which one of the domains best represents each OHIP question?*” and the descriptive statistics including each item’s CVI value, *K*_*free*_, and levels of agreement.

**Table 4:**
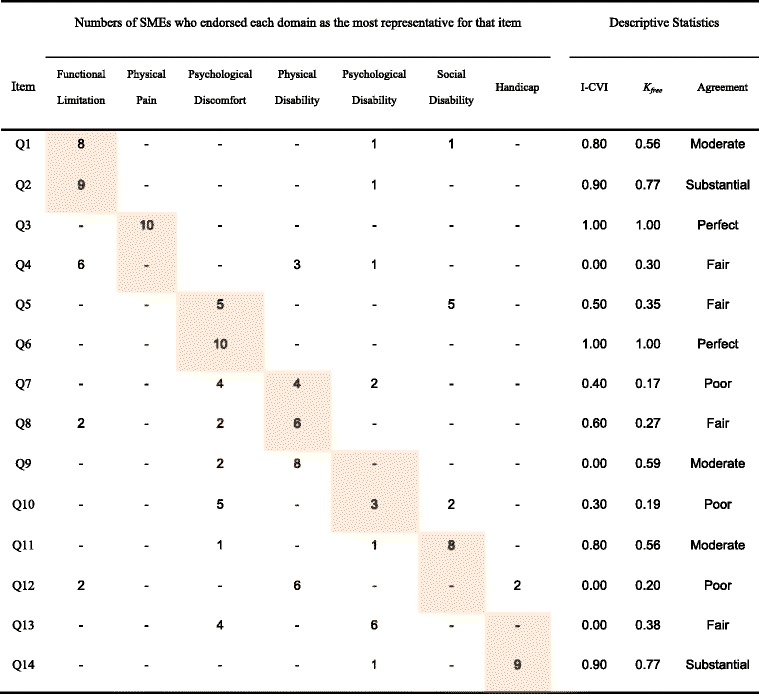
The SMEs’ classification of items into the seven OHIP domains, Item-level Content Validity Index (I-CVI), Free-Marginal Kappa (*K*_*free*_), and levels of agreement on the Relevance Index

*Note:* Shaded cells indicate the theoretical domains predetermined by Slade and Spencer (1994).

Examination of each question’s relevance to the expected theoretical domain revealed that the endorsement rates for questions 1, 2, 3, 6, 11, and 14 were equal to or above the acceptable CVI value of 0.80 (S-CVI_relevance_ = 0.42, *K*_*free*_ > 0.4), confirming that the questions corresponded with the hypothesized domains by the Locker’s model. On the other hand, eight out of 14 questions (# 4, 5, 7, 8, 9, 10, 12, and 13) felt below the acceptable CVI value, with 5 showing poor or fair agreement (*K*_*free*_ < 0.4). Closer examination of content-invalid items revealed that question 5, “*self-conscious*” (psychological discomfort); question 9, “*difficult to relax*” (psychological disability); and question 13, “*life in general was less satisfying*” (handicap) were also representing social disability (*K*_*free*_ = 0.4), physical disability (*K*_*free*_ = 0.6), and psychological disability (*K*_*free*_ = 0.4), respectively. Poor agreement was noted for questions 7, 10, and 12 (*K*_*free*_ < 0.2) while questions 4 and 8 showed fair agreement (0.2 < *K*_*free*_ < 0.4). Overall, 7 SMEs indicated that some questions could be interpreted outside of the oral health context.). For example, question 12, “*difficulties in doing one’s usual jobs*,” may unintentionally elicit non-dental-related experiences if left ‘as is’. The seemingly transferrable construct of OHQoL can be understood differently in English and in non-western cultures such as Korean due to differences in priorities, health perceptions and potential impact of a disorder. Another SME indicated that OHIP-14 K does not adequately capture the aesthetic concerns that patients might have about their mouths. In their overall evaluation of OHIP-14 K, 6 SMEs recognized the need for better accuracy of the OHIP-14 K items to ensure cross-cultural equivalence.

## Discussion

Our study employed SMEs to culturally assess the content and equivalence of an OHRQoL instrument to Korean. As advised by Sischo and Broder (2011), OHQoL has multiple applications in dental research and services especially when we move from a bench research to a more person-centered approaches to measure treatment needs and efficacy of care [22]. In turn, the availability of cross-culturally valid and reliable OHQoL measures is beneficial for needs assessment, oral health care planning, and service evaluation in Korean as well as in other languages. Despite the widespread use of OHIP in English and in more than 30 different languages, content validity and equivalency of its translated versions – including the OHIP-14 K – have not been fully addressed [6]. This was compounded by the fact that current cultural adaptation and validation strategies using the suggested forward and backward translations supervised by a committee are not resistant to biases; ramifications to inferences made on cultural differences in OHQoL are expected. Typical validation efforts for the OHIP use criterion-related approaches that are vulnerable to the cross-cultural biases and misunderstandings while paying little attention to the content of the scale or theoretical foundations. Consequently, the validity of the OHIP translated to other languages and applied to other cultures needs a critical discussion [5] since none of the existing translations of the OHIP, including the Korean, seems to challenge Locker’s ICIDH concept of disturbances to oral health-related quality of life [6].

In contrast to the traditional committee method of establishing equivalency [23–25], our study employed SMEs who investigated the theoretical foundations of the OHIP-14 K and recommended changes. The scale-level CVIs obtained in our study demonstrated to be an alternative method for content validation and indicated that wording of the OHIP-14 K is clear (S-CVI_clarity_ = 0.93), but it may not be cross-culturally equivalent to its English counterpart (S-CVI_equivalence_ = 0.50). The positive results on the clarity index were expected considering that OHIP-14 K has already undergone rigorous testing with monolingual Korean adults and five Korean dentists [15] despite its cultural equivalency never being fully established. Our results also indicated a limited degree of relevance for the entire scale (S-CVI_relevance_ = 0.42). Cross-culturally valid scales must demonstrate evidence of item relevance and mutual exclusiveness of their theoretical dimensions to achieve proper representation of the construct [26]. For this reason, each of the seven domains should be represented by at least two content-valid items to avoid taking chances with translation quality. Only half of 14 OHIP-14 K questions accurately loaded onto the expected seven domains as proposed by Slade and Spenser, while John and colleagues employed exploratory factor analysis to determine that the OHIP lends itself to four, not seven domains [14]. The low level of relevance of the set of assigned domains can imply a departure from the original conceptual model and an inaccurate representation of the construct since the subscales are not measuring what they are supposed to measure. This finding also raises questions about the use of subscale scores as a valid and reliable indicator of OHQoL domains while little attention has been placed to discuss responsiveness to changes of scores from the translated OHIP in the clinical status of respondents [5, 14].

The scale elements met the minimally acceptable disagreement level (AD_M_ ≤ 0.56 or *K*_*free*_ > 0.40) but were below the acceptable 0.8 value of CVI and needed to be revised or eliminated according to the experts’ suggestions. However, instead of eliminating items from an already short scale, revisions of the OHIP-14 K instructions, response format and frequency as well as questions 5, 6, and 9 were suggested (Table 5). Standardizing the recall periods is necessary to minimize construct-irrelevant variance for making valid cross-cultural comparisons. In addition, the inclusion of the phrase “*because of problems with your teeth, mouth or dentures*”, omitted in the Korean version [15], would help respondents to focus on oral health-related events when answering the questions.

**Table 5:**
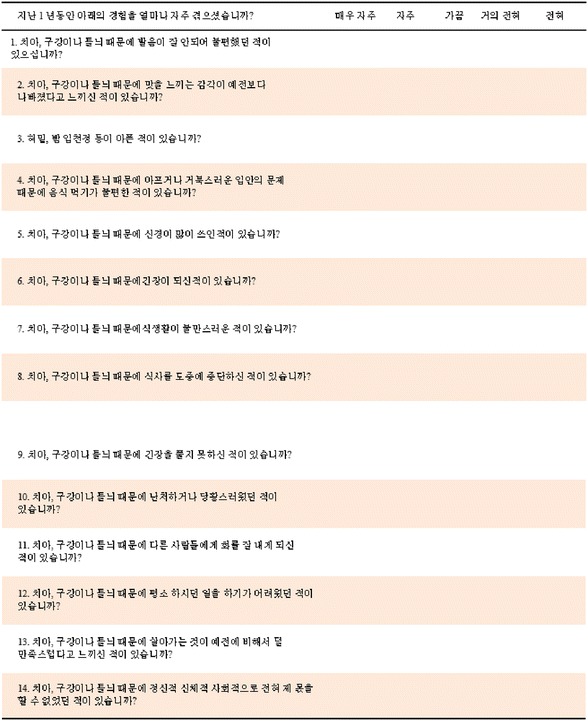
Suggested revised version of OHIP-14 K

In the literature, various types of biases have been reported in other language versions of the OHIP. When question 3, “*have you had any painful spots or areas in your mouth?*” was translated into Brazilian Portuguese, it used the word “*pontos*” for spot. However, the word also has a second meaning – suture stitches – which caused confusion among the respondents [27]. A similar translation problem was reported by Kenig and Nikolovska (2012) in the Macedonian version of the OHIP item 5, “*self-consciousness*”[28]. In Macedonian, the literal translation of the term had a different meaning than intended whereas in the Korean version, the low degrees of translation equivalence (I-CVI = 0.4) and relevance (I-CVI = 0.5) suggest that the translation may have been too liberal. In the case of the Macedonian version, the authors decided to eliminate the item because most respondents did not understand its meaning [29].

There are a number of possible explanations for the reported translation problems. As concluded by Guillemin et al. [12, 25], there is no “standardized approach to the cross-cultural adaptation of HRQOL instruments” which probably corroborates the fact that there is no detailed explanation of how conceptual equivalence has been indeed explored within the OHIP [14]. Another source of asymmetry could have been the ambiguities in the English version itself. As discussed previously, the *handicap* domain included questions that were artificially added to the original OHIP and not directly developed from the interviews with the lay Australian respondents [8]. In the case of our study, none of the SMEs judged question 13, “*life less satisfying*,” to represent the handicap domain. Moreover, disagreement was noted for question 14, “*totally unable to function*,” which was translated to signify “*a state of being incapacitated psychologically, physically and socially*”. Not only can such a triple-barrelled question be confusing for respondents, but it also gives a very vague impression of “handicap” for which no equivalent word exists in Korean. Cultural equivalence aside, these two questions were loaded into psychosocial impact, one of the four domains identified by John et al. to better structure the OHIP and to be ‘similar across cultures and populations’ [14]. Likewise, our study found that seemingly equivalent items did not always guarantee their relevance to the expected theoretical domains. For example, although question 9, “*have you had difficulties relaxing?*” was judged to be equivalent to its English counterpart (I-CVI_equivalence_ = 0.8), it did not load onto the expected *psychological disability* domain (I-CVI_relevance_ = 0.0). A similar translation issue was reported by Room and colleagues (1996), who noted that Korean translations of psychological affective states were easily mistaken for physical states because Korean words regarding feelings do not effectively differentiate between physical sensations and emotions [30].

Finally, there could have been conceptual differences across cultures in interpreting the semantic equivalence as advised by Herdman [31] who highlighted that translated instruments implicitly and explicitly assume that notions of oral health-related quality of life are similar across cultures, when they may not be. Hence, while revisions of the questions are critical to the validity of cross-cultural comparisons of OHQoL, conceptual equivalence remains the most challenging part of the translational process and it has been either addressed superficially or omitted altogether from many of the OHIP translations [6].

### Implications of the Content Validity Findings

Our study found limited evidence of content validity for OHIP-14 K in terms of relevance and cultural equivalency with the English version. In line with previous research [32, 33], the SMEs’ suggestions for improving the scale’s content validity underscored the importance of its cultural appropriateness and faithfulness to the source version and theoretical mode. Our study suggests that OHIP-14 K should have the same relationship with the construct of interest both within and across cultural groups.

Another implication of our study includes the impact of the detected biases on cross-cultural comparisons of OHQoL. The same degree of construct might elicit different responses on a Likert scale and consequently biased interpretation of domain and total scores [34]. For example, a physical disability domain could be measuring social disability, or could be loaded into a structured factor to better characterise OHQoL across cultures [14]. Hence, the fragile relationship between items and their theoretical domains carries significant implications as OHIP scores are calculated for each domain as well as for the entire scale [5]. However, Brondani and MacEntee raised a more fundamental problem with the use of a summative score due to the questionable discreteness and stability of the theoretical domains of Locker’s model. While John et al. (2014) suggested four factors to better structure the OHIP domains [14], Bakers (2007) questioned whether or not the OHIP domains were actually distinguishable [35] and if so, how they would readily relate to one another as per Locker’s conceptual framework.

Based on the findings presented here, a refined version of OHIP-14 K was yielded to enhance both semantic and conceptual equivalence (Table 5). Although more work is needed to evaluate its psychometric properties in a target Korean sampling group, it emerged based on the assumption that the existing OHIP Korean translation holds validity. Although there is no question that the OHIP is a psychometric instrument tested and widely used, there are other techniques to provide insight about the OHIP's ability to measure invariance across populations.

Limitations of our study include the use of a convenient sample of dentists who suggested changes in the OHIP as an exercise of content validity and cultural equivalency. Multi-disciplinary SMEs from other disciplines could have provided a more diverse range of knowledge and experience as conducted by others [17, 36]. The CVI technique used should be supplemented with other validation testing specially within the target population. Likewise, the proposed OHIP version on Table 5 remains critical to be consulted by lay Koreans who could examine the relevance and utility of the OHIP-14 K revisions suggested in this study. In turn, no data exists to support a better scoring system for validity, reliability and responsiveness in the proposed modified version of the OHIP to the target population.

## Conclusion

Like the OHIP, patient-oriented outcome measures can only enhance our appreciation for the relationships between oral and general health across cultures once they are indeed assessing the same domains and constructs of interest within a content valid and culturally equivalent measure. Our study showed that:

- ➣ The CVI technique is an alternative tool for evaluating content validity and equivalency of an OHQoL measure and documenting the content validation process and quantification of CVIs and disagreement indices.
- ➣ The expert suggestions and the CVI ratings could be used also to improve the content validity and equivalency of the OHIP-14 K, as well as to refine inferences made from cross-cultural measurements of OHQoL.
- ➣ The OHIP-14 K demonstrated limited evidence of content validity and cultural equivalency, and potential cross-cultural biases have been identified in its method, items, and construct representation.
- ➣ Future studies should be done to establish content validity and cultural equivalency of other language versions of OHIP-14 (or other OHQoL scales) and further explore the utility of CVIs and disagreement indices as there is a need for a continuous evaluation of the scale for the intended target populations.
